# Life satisfaction data in a developing country: CaliBRANDO measurement system

**DOI:** 10.1016/j.dib.2017.06.038

**Published:** 2017-06-28

**Authors:** Lina Martínez

**Affiliations:** Universidad Icesi, Observatorio de Políticas Públicas–POLIS, Cali, Colombia

**Keywords:** Life satisfaction, Government performance, Colombia, South America

## Abstract

This paper describes one large multi-annual research project–CaliBRANDO–about subjective wellbeing in a developing country. CaliBRANDO is a life satisfaction measurement system implemented in Cali, the third largest city in Colombia, South America. Data have been collected annually since 2014 and aim at collecting comprehensive temporal information about individual-level subjective wellbeing and its relationship with government performance. CaliBRANDO is the only study in Colombia that measures subjective wellbeing in a large city in this way. This paper presents the methodology followed in the study and discusses the relevance of the data collected.

**Specifications Table**

**Table:** 

Subject area	Public policy
More specific subject area	*Subjective wellbeing*
Type of data	Text, dummy, and metric variables
How data were acquired	Population survey
Data format	Raw
Experimental factors	None
Experimental features	None
Data source location	Cali –Colombia
Data accessibility	Observatorio de Políticas Públicas–POLIS www.icesi.edu.co/polis/
Related research article	POLIS (2017) CaliBRANDO measurement system. Policy brief No. 17

**Value of the data**

• CaliBRANDO data allow measurement and tracking of individual life satisfaction ratings in a large metropolitan area in Colombia. Data collected are comparable with international data about subjective wellbeing.
• It is possible to link life satisfaction with government performance in several domains. Data also allow the construction of valid indexes to proxy government performance and citizens’ perceptions in different domains.
• Data allow the establishment of individual factors that affect life satisfaction. Information at the individual level includes variables such as education, income, employment, savings, health condition, and living standards.
• CaliBRANDO system gathers data about health condition. It uses a widely known health index (CDC5) which proxies for perceived general health condition and number of days feeling physically or mentally unwell. The survey also measures weight, height, and abdominal circumference of each respondent to facilitate the reporting of reliable data on overweight people and obesity rates. All health measures are comparable with international data.

## 1 Data

CaliBRANDO is a survey conducted annually by the Observatory of Public Policies (POLIS) of Universidad Icesi since 2014. This survey measures life satisfaction and is the only study in Colombia created with the main objective of measuring subjective wellbeing. CaliBRANDO is representative in terms of the city´s gender distribution, socioeconomic strata and race/ethnicity composition. Surveys are conducted via face-to-face interviews with adults (18 and older) by trained pollsters in 53 locations across the city. To ensure data quality, during fieldwork there are four pollsters’ supervisors present. Informants are randomly selected. Respondents are approached by explaining the objective of the study, assuring confidentiality, and emphasizing that the data will be used for academic purposes. In addition, it is made clear to respondents that they could stop the survey at any time and participation is voluntary. Respondents are measured during the survey in terms of their weight, height and abdominal circumference. For this purpose, each pollster has an electronic scale and a meter tape.

CaliBRANDO uses a stratified multi-stage sampling system; every year about 1200 surveys are completed. Information is collected in eight areas: sociodemographic information, life satisfaction, educational attainment and expectations, employment and job quality, income and living standards, health, satisfaction with government performance, and satisfaction with personal domains. The next section explains each area in detail.

This study follows local and international rules for empirical research and is approved by the Institutional Review Board of Universidad Icesi. Likewise, respondents provide verbal consent before survey commencement. CaliBRANDO data are available at: www.icesi.edu.co/polis/. There is a yearly policy brief displaying principal findings, available in Spanish and English [1].

CaliBRANDO data are used for three purposes:

1. Produce academic research.
2. Generate a yearly policy brief aimed at disseminating academic research on life satisfaction to a broader array of stakeholders beyond academia, including the public.
3. Provide information to local government about life satisfaction and its relationships with government performance.

## 2 Experimental design, materials and methods

The CaliBRANDO survey is structured in eight sections. Each section is composed of different questions that facilitate the creation of composite indexes. The structure presented below (Table 1) reflects the 2016 survey when new questions were added compared to the 2015 and 2014 versions of the questionnaire.

**Table 1:** CaliBRANDO survey structure.

**Sociodemographic information**	Year of birth
	Neighborhood of residence
	Gender
	Socioeconomic strata of household^a^
	Race/ethnicity
	Literacy
	Educational attainment
	Number of people in the household
	Household role or position
	Have children
	Number of children
	Age of first child
	Plan on having more children
	Marital status
	Living in rented or paid household
	Ownership of any type of property
	Owns a motorcycle or car
	Type of public transportation used the most
	Average income
**Life satisfaction**	On a scale of 1–10, 1 being the lowest and 10 the highest, how satisfied are you living in the city?
	On a scale of 1–10, 1 being the poorest area and 10 the richest, where do you consider your household is located?
	On a scale of 1–10, 1 being the lowest and 10 the highest, how satisfied are you with your life?^b^
	What do you need to be completely satisfied with your life?
**Educational attainment and expectations**	Currently studying
	Type of study
	Plan to continue studying
	Reasons for not continuing studying
**Employment and job quality**	Main economic/labor activity
	Unemployment
	Average hours worked last week
	Satisfied with current occupation/job
	Type of job contract
	Satisfied with type of job contract
	Contribution to health and retirement
	Benefits under current job contract
**Income and living standards**	Poverty perception
	Perception of economic improvement in household during last year
	Perception of being better off economically in the near future
	Perception of being better off economically than parents
	Satisfied with living standards
	What is necessary to be completely satisfied with your living standards
	Have savings to live at least three months in case of unemployment
	Required income to live without economic hardships
	Actions taken to ensure a stable retirement
**Health**	General health condition
	Number of days during last month that physical health was not good
	Number of days during last month that mental health was not good
	Height
	Weight
	Satisfied with current weight
	Abdominal circumference
	Physical activity – exercise
	Frequency of physical activity – exercise
	
**Satisfaction with government performance**	On a scale of 1–10 how satisfied are you with government performance in the following areas:
	Security
	Health services
	Public transportation
	Employment generation
	Parks and public spaces
	Education
	Public services
	Traffic
	Neighborhoods
**Satisfaction with personal factors**	On a scale of 1–10 how satisfied are you with the following personal domains*:*
	Family
	Employment
	Emotional life
	Health
	Household economy
	Income
	Education
	Place of living

a In Colombia, households are classified into socioeconomic strata on a scale of 1–6. One represents the poorest, six the richest. This classification helps determine households with similar social and economic features, according to the external physical characteristics of the household and its environment. This information is then used to direct welfare policies.

b In 2017 the answer scale for this question was changed to 0–10 to facilitate international comparisons.

## Figures and Tables

**Table:** 

**CaliBRANDO descriptive statistics 2014–2016**				
	**2014**	**2015**	**2016**	**Average 2014–2016**
**Gender**				
Men	50,2%	49,7%	49,4%	49,9%
Women	49,8%	50,3%	50,1%	50,1%
**Average age (years)**				
Men	40	39	36	38
Women	38	37	36	37
**Educational attainment**				
Elementary school	10,2%	10,0%	11,9%	10,7%
High school	40,4%	48,2%	46,8%	45,1%
Any level of higher education	49,5%	41,8%	41,4%	44,2%
**Income**^a^				
Less than the minimum monthly wage	22,9%	27,8%	25,1%	25,3%
Between 1 and 2 × minimum monthly wage	51,6%	56,0%	54,0%	53,9%
Between 2 and 4 × minimum monthly wage	17,8%	11,0%	14,3%	14,6%
More than 4 × minimum monthly wage	7,8%	5,3%	6,5%	6,5%
**Average monthly income (US dollars)**	$ 385	$ 343	$ 364	$ 363,00
**Employment**				
Formal employment	53,79%	49,07%	50,68%	51,2%
Informal employment	46,21%	50,93%	49,32%	48,8%
**Marital status**				
Single	44,14%	47,46%	50,30%	64,3%
Married/cohabitation	49,39%	44,32%	43,16%	44,6%
Divorced	6,47%	8,22%	6,54%	9,6%
**Socioeconomic strata**^b^				
Low (poverty)	49,83%	51,04%	50%	50,3%
Medium (middle class)	40,13%	39,47%	40,05%	39,9%
High (rich)	10,03%	9,49%	9,95%	9,8%
**Has children**				
Men	45,88%	45,74%	44,83%	57,30%
Women	54,12%	54,13%	55,17%	68,40%
**Life satisfaction score (1–10 scale)**	8,3	8,7	8,5	8,6
**Obs**	**1206**	**1204**	**1206**	**3616**

a Minimum monthly wage = US245 dollars. 1 US Dollar = 3000 Colombian pesos.

b Government scale to classify households.

## References

[1] Observatorio de Políticas Públicas–POLIS. Life satisfaction: measurement and its implications for public policy formulation. POLIS Policy Brief, Universidad Icesi, Cali–Colombia, 17, 2017, pp. 5–21.

