# Supplementary material for: Life satisfaction data in a developing country: CaliBRANDO measurement system
Source: Data Brief. 2017 Jun 28;13:600–4. doi: 10.1016/j.dib.2017.06.038 (PMC5501882; doi:10.1016/j.dib.2017.06.038)
Supplement: Supplementary file 1 — Supplementary material [file mmc1.pdf]

Conflicts of Interest Statement

Manuscript title: **Life Satisfaction Data in a Developing Country: CaliBRANDO Measurement System**

The authors whose names are listed immediately below certify that they have NO affiliations with or involvement in any organization or entity with any financial interest (such as honoraria; educational grants; participation in speakers' bureaus; membership, employment, consultancies, stock ownership, or other equity interest; and expert testimony or patent-licensing arrangements), or non-financial interest (such as personal or professional relationships, affiliations, knowledge or beliefs) in the subject matter or materials discussed in this manuscript.

Author names:

Lina Martínez

This statement is signed by all the authors to indicate agreement that the above information is true and correct

Name	Signature	Date
Lina Martínez	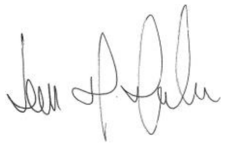	April 27 - 2016
