# Supplementary material for: Life satisfaction data in a developing country: CaliBRANDO measurement system
Source: Data Brief. 2017 Jun 28;13:600–4. doi: 10.1016/j.dib.2017.06.038 (PMC5501882; doi:10.1016/j.dib.2017.06.038)
Supplement: Supplementary file 2 — Supplementary material [file mmc2.pdf]

UNIVERSIDAD ICESI — POLIS — Cali BRANDO 2016

Fecha aplicación:	Mes		Día		Zona de realización de la encuesta:
Nombre del Encuestador(a):					Revisado por:

DATOS DEMOGRÁFICOS

1.¿Usted es residente de Cali?

1 [ ] Sí

0 [ ] No *(Termine la encuesta)*

2.Año de nacimiento: ____________

(Solo mayores de edad, personas nacidas antes de 1998)

3. ¿En qué barrio vive? ____________

4. Sexo

1 [ ] Hombre
2 [ ] Mujer
3 [ ] Intersexo

5. ¿Cuál es el estrato socio-económico de su vivienda?

1 [ ] 2 [ ] 3 [ ] 4 [ ] 5 [ ] 6 [ ]

6.1 ¿Cuál es el nivel educativo más alto alcanzado por usted (así no lo haya terminado) y el último grado aprobado en este nivel?

*Marque con una X el nivel educativo y escriba el último grado o año en ese nivel

		(1)Nivel	(2)Año
1	Primaria incompleta		
2	Primaria completa		
3	Secundaria incompleta		
4	Secundaria completa		
5	Técnica/Tecnológica*		
6	Profesional*		
7	Especialización*		
8	Maestría/Doctorado*		
9	Ninguno		NA
10	No sabe		NA

***Especifíque si son semestres o años**

6.2 ¿Cuántos años de educación recibieron sus padres?

1. Padre ________ 99 [ ] No sabe

2. Madre ________ 99 [ ] No sabe

7. De acuerdo con su cultura, pueblo o rasgos físicos usted se reconoce como:

1 [ ] Blanco
2 [ ] Mestizo
3 [ ] Indígena
4 [ ] Negro/Afro
5 [ ] Otro
6 [ ] Ninguno

8. ¿Cuál es su estado civil?

1 [ ] Casado(a)
2 [ ] Unión Libre
3 [ ] Soltero(a)
4 [ ] Separado/ Divorciado
5 [ ] Viudo

9. Su tipo de vivienda es:

1 [ ] Propia pagando
2 [ ] Propia pagada
3 [ ] Arrendada
4 [ ] Familiar
5 [ ] Otra, ¿cuál? ____________

10. En su hogar, ¿que posición ocupa?

1 [ ] Jefe de hogar
2 [ ] Hijo(a)
3 [ ] Ama de casa
4 [ ] Otro

11. Incluyéndolo a usted, ¿cuántas personas viven en su hogar? ____________

(N° de personas con las que comparte alimentos)

12. En su hogar, ¿hay personas en edad de trabajar?

1 [ ] Sí
- 1.1 ¿Cuántas tienen empleo? ______
- 1.2 ¿Cuántas están buscando empleo? ______

2 [ ] No *(Pase a la pregunta 13)*

13. ¿Cuántas personas aportan recursos económicos para el sustento de su hogar? ____________

14. ¿Usted tiene hijos?

1 [ ] Sí
- 1.1 ¿Cuántos? ________
- 1.2 ¿A qué edad tuvo su primer hijo? ______
- 1.3 ¿Quisiera tener más hijos? [ ] Sí 0 [ ] No

2.1 ¿Planea o desea tener hijos en el futuro?

2 [ ] No → 1 [ ] Sí 0 [ ] No

15.En promedio, ¿a cuánto ascienden sus ingresos mensuales?

1 [ ] Menos de 1 smlv (<689.454)
2 [ ] Entre 1 smlv y menos de 2 smlv (689.454 - 1'378.908)
3 [ ] Entre 2 smlv y menos de 4 smlv (1'378.908 - 2'.757.816)
4 [ ] Entre 4 smlv y menos de 8 smlv (2'.757.816 - 5'515.632)
5 [ ] Más de 8 smlv (>5'515.632)
6 [ ] No tiene ingresos
7 [ ] No sabe/no responde

16. ¿Usted es propietario de algún bien raíz? Ej.: Casa, apartamento, finca, tierra

1 [ ] Sí
0 [ ] No

17. ¿Tiene medio de transporte motorizado propio?

1 [ ] Sí → *Indique el tipo, cantidad y modelo
0 [ ] No *(Pase a la p18)*

	(1) Tipo	(2)Cantidad	(3) Modelo*
1	Moto		
2	Carro		

*** Si tiene más de un vehículo, registre solamente el modelo más reciente**

18. ¿Qué medio de transporte utiliza con más frecuencia para desplazarse en Cali? (ÚNICA RESPUESTA)

1 [ ] Taxi
2 [ ] MIO/ Bus
3 [ ] Bicicleta propia
4 [ ] Pirata/motoratón
5 [ ] Vehículo propio
6 [ ] Otro, ¿Cuál? ____________

19. En promedio, ¿cuánto tiempo toman sus desplazamientos diarios para desarrollar su actividad principal?

1.Ida ____________
2. Regreso ____________

3. Frecuencia
1 [ ] Hora(s)
2 [ ] Minutos

SATISFACCIÓN CON LA VIDA Y LA CIUDAD

20. En una escala de uno a diez, califique qué tan satisfecho se siente con Cali

1	2	3	4	5	6	7	8	9	10

21. En una escala de uno a diez, donde 1 son los más pobres de la ciudad y 10 los más ricos, ¿en dónde se sitúa su hogar?

1	2	3	4	5	6	7	8	9	10

22. En una escala de uno a diez, califique qué tan satisfecho se siente con su vida (SI CONTESTA 10 PASE A LA PREGUNTA 24)

1	2	3	4	5	6	7	8	9	10

23.¿Qué hace falta para que esté satisfecho con su vida? (RESPUESTA ESPONTÁNEA - MÚLTIPLE, MÁXIMO 3 OPCIONES, *NUMERAR ORDEN DE RESPUESTA*):

( ) 1 [ ] Buena salud
( ) 2 [ ] Plata
( ) 3 [ ] Casa propia
( ) 4 [ ] Pareja estable
( ) 5 [ ] Trabajo
( ) 6 [ ] Familia
( ) 7 [ ] Mejores relaciones familiares
( ) 8 [ ] Estudio
( ) 9 [ ] Éxito personal/profesional
( ) 10 [ ] Mejorar aspecto físico
( ) 11 [ ] Tiempo libre
( ) 12 [ ] Carro/medio de transporte propio
( ) 13 [ ] Seguridad
( ) 14 [ ] Salud de un familiar
( ) 15 [ ] Otro, ¿Cuál? ____________

UNIVERSIDAD ICESI · POLIS · Cali BRANDO 2016

EDUCACIÓN

24. ¿En la actualidad está recibiendo algún tipo de capacitación o estudio formal?

1 ☐ Sí
0 ☐ No *(Pase a pregunta 26)*

25. ¿Qué tipo de estudio está realizando?

1 ☐ Primaria
2 ☐ Secundaria
3 ☐ Técnica
4 ☐ Profesional
5 ☐ Especialización/Diplomado
6 ☐ Maestría/Doctorado
7 ☐ Capacitación laboral
8 ☐ Otro, ¿Cuál? ____________________

26. ¿Quisiera seguir estudiando?

1 ☐ Sí
0 ☐ No *(Pase a la sección Trabajo)*

27. ¿Planea seguir estudiando en el corto plazo (6 meses)?

1 ☐ Sí *(Pase a la sección Trabajo)*
2 ☐ No
3 ☐ No sabe/no responde

28. ¿Por qué no? - RESPUESTA ESPONTÁNEA (ÚNICA RESPUESTA)

1 ☐ Por falta de recursos
2 ☐ No le gusta estudiar
3 ☐ Obligaciones económicas
4 ☐ Falta de tiempo
5 ☐ Porque tengo familia
6 ☐ No es lo mío
7 ☐ Otro, ¿Cuál? ____________

TRABAJO

29. ¿En qué actividad ocupó la mayor parte del tiempo la semana pasada?

1 ☐ Trabajando en una empresa - *(Pase a la pregunta 31)*
2 ☐ Trabajando como independiente - *(Pase a la p31)*
3 ☐ Buscando trabajo - *(Pase a la pregunta 30)*
4 ☐ Estudiando - *(Saltar sección)*
5 ☐ Oficios del hogar - *(Pase a la pregunta 39)*
6 ☐ Ninguna actividad en especial - *(Pase a la p39)*
7 ☐ En vacaciones del trabajo - *(Pase a la pregunta 32)*
8 ☐ En vacaciones académicas - *(Saltar sección)*
9 ☐ Otro, ¿Cuál? ____________

30. ¿Cuánto tiempo lleva desempleado? - SÓLO PARA DESEMPLEADOS

____________ **(MESES)**

(Pase a la pregunta 39)

31. ¿Cuántas horas trabajó la semana pasada?

____
Número de horas

32. ¿Se encuentra conforme con su ocupación?

1 ☐ Sí
2 ☐ No
3 ☐ No sabe/no responde

33. ¿Cuánto tiempo lleva en su trabajo actual?

____
(Indicar Meses o Años)

34. ¿Ha recibido capacitaciones de su trabajo en el último año?

1 ☐ Sí
0 ☐ No

35. ¿Cree que tendrá trabajo asegurado en los próximos 6 meses?

1 ☐ Sí
0 ☐ No

36. ¿Cuáles son los beneficios no salariales más importantes que usted obtiene de su trabajo? (MÁXIMO TRES OPCIONES)

1 ☐ Horario flexible
2 ☐ Auxilio educativo
3 ☐ Gimnasio
4 ☐ Salud (medicina prepagada/seguro)
5 ☐ Servicio de telefonía celular
6 ☐ Casino / Vales de comida
7 ☐ Crédito con intereses bajos
8 ☐ Transporte (bus/vehículo/vale gasolina)
9 ☐ Descuentos
10 ☐ Ninguno
11 ☐ Otro ¿Cuál? ____________

37. ¿Tiene contrato verbal o escrito? - *NO APLICA A INDEPENDIENTES*

1 ☐ Contrato Verbal
2 ☐ Contrato Escrito
3 ☐ No aplica *(Pase a la p39)*
4 ☐ No sabe/no responde *(Pase a la p39)*

38. ¿Está conforme con el contrato que tiene?

1 ☐ Sí
2 ☐ No
3 ☐ No sabe/no responde

39. ¿Usted cotiza a salud y a pensión?

1 ☐ Sólo a salud
2 ☐ Sólo a pensión
3 ☐ Ambas
4 ☐ Ninguno
5 ☐ Es pensionado(a)
6 ☐ No sabe/no responde

INGRESO

40. ¿Podría decirme si usted se considera pobre?

1 ☐ Sí
2 ☐ No
3 ☐ No sabe/no responde

41. ¿Usted diría que en su hogar se encuentran mejor o peor económicamente que lo que estaban hace un año?

1 ☐ Mejor
2 ☐ Igual
3 ☐ Peor
4 ☐ No sabe/no responde

42. Mirando hacia el futuro, ¿usted cree que dentro de un año su hogar va a estar mejor, peor o igual económicamente?

1 ☐ Mejor
2 ☐ Igual
3 ☐ Peor
4 ☐ No sabe/no responde

43. Comparándose con sus padres a su misma edad, ¿usted ha mejorado o empeorado en su condición socio-económica?

1 ☐ Mejorado
2 ☐ Sigue igual
3 ☐ Empeorado
4 ☐ No sabe/no responde

Pase a la pregunta 45 (opciones 2–4)

44. ¿Cuál es la principal razón por la que usted cree que su situación socioeconómica ha mejorado respecto a la situación de sus padres? (ÚNICA RESPUESTA)

1 ☐ Mayores niveles educativos
2 ☐ Mayores tasas de empleo en el sector formal
3 ☐ Familia más pequeña
4 ☐ Más suerte que sus padres
5 ☐ Vivir en la zona urbana
6 ☐ No sabe/no responde
7 ☐ Otra, ¿cuál? ____________

45. ¿Usted está satisfecho con su estándar de vida? Es decir, con todas las cosas que puede comprar y hacer (Gallup World Poll)

1 ☐ Sí *(Pase a la pregunta 47)*
2 ☐ No *(Pase a la pregunta 46)*
3 ☐ No sabe/no responde *(Pase a la pregunta 47)*

46.¿Qué le hace falta para estar satisfecho(a) con su estándar de vida? (ÚNICA RESPUESTA)

1 ☐ Pagar deudas
2 ☐ Casa propia
3 ☐ Más plata
4 ☐ Estudiar
5 ☐ Viajar
6 ☐ Ahorrar
7 ☐ Vacaciones
8 ☐ Trabajo
9 ☐ Otro ¿Cuál? ____________

47. ¿Durante el último año, usted o alguien de su hogar ha realizado un gasto o inversión importante (como la compra de un vehículo o electrodoméstico, viaje)? (OPCIÓN MÚLTIPLE)

1 ☐ Compra de vehículo
2 ☐ Compra de electrodoméstico
3 ☐ Viaje
4 ☐ Compra de bien raíz
5 ☐ Ninguno
6 ☐ Otro ¿Cuál? ____________

48. ¿Tiene usted ahorros suficientes para vivir al menos tres meses, en caso de quedarse sin trabajo? - *NO APLICA A ESTUDIANTES, AMAS DE CASA NI PENSIONADOS*

1 ☐ Sí
2 ☐ No
3 ☐ No sabe/no responde
4 ☐ No aplica

49. ¿Qué ha hecho durante su vida para asegurarse en el futuro una buena vejez? (OPCIÓN MÚLTIPLE) - *NO APLICA A ESTUDIANTES*

1 ☐ Le apuesta a los hijos para que ellos le ayuden
2 ☐ Guarda un dinero para el futuro
3 ☐ Monta o trata de organizar un negocio/activo que le garantice una renta
4 ☐ Cotiza en un fondo de pensiones
5 ☐ Espera que algún día le alcance la plata para guardar para la vejez
6 ☐ Nada
7 ☐ Otro, ¿cuál? ____________

50. Dadas las condiciones económicas actuales de su hogar, cuánto considera usted que sería un ingreso mensual en su hogar:

1. Insuficiente (Alcanza sólo para cubrir necesidades básicas del hogar) $ ____________
2. Bueno (Cubre todas las necesidades básicas del hogar y sobra) $ ____________
3. Muy bueno (Permite vivir con comodidad y ahorro) $ ____________

SALUD

51. ¿ A cuál régimen de seguridad social en salud está afiliado?

1 ☐ Cotizante → 1.1 ☐ Prepagada
2 ☐ Subsidiado → 2.1 ☐ EPS
3 ☐ Beneficiario
4 ☐ Especial
5 ☐ Ninguno
6 ☐ No sabe/no responde

52. Usted diría que, en general, su salud es - (CDC 5):

1 ☐ Excelente
2 ☐ Muy buena
3 ☐ Buena
4 ☐ Regular
5 ☐ Mala
6 ☐ No sabe/no responde

53. Ahora piense acerca de su salud física, la cual incluye enfermedades físicas y accidentes, ¿Durante cuántos de los pasados treinta días no gozó de buena salud física? - (CDC 5)

Número de días ____________

99 ☐ No sabe/No responde

54. Ahora piense acerca de su salud mental, la cual incluye tensión, depresión y problemas emocionales: ¿Durante cuántos de los pasados treinta días no gozó de buena salud mental? - (CDC 5)

Número de días ____________

99 ☐ No sabe/No responde

55. ¿Durante cuántos de los pasados treinta días, el mal estado de salud mental o física le impidieron realizar sus actividades, tales como cuidado personal, trabajo o recreación? - (CDC 5) - *SI EN LAS DOS PREGUNTAS ANTERIORES CONTESTÓ "NINGUNO" SALTAR ESTA .*

Número de días ____________

99 ☐ No sabe/No responde

UNIVERSIDAD ICESI — POLIS — Cali BRANDO 2016

CONTINUACIÓN

56. Ahora le voy a hacer unas preguntas sobre su fisionomía.

1. ¿Cuál es su altura? ____________
2. ¿Cuál es su peso? ____________
3. ¿Se siente conforme con su peso actual? → 1 [ ] Sí 0 [ ] No
4. Circunferencia abdominal __________

57. ¿Realiza alguna actividad física como trotar, caminar, practicar algún deporte o ir al gimnasio?

1 [ ] Sí → ***Indique la frecuencia y el número de dias**

0 [ ] No *(Pase a la p58)*

	1. Frecuencia	2. N° dias
1	Semanal	
2	Mensual	

OTROS

58. En general, ¿se siente seguro en la ciudad?

1 [ ] Sí
0 [ ] No

59. Durante el último año, ¿usted o algún miembro de su hogar ha sido víctima de robo, homicidio, violencia física/sexual, secuestro o extorsión?

	(1) Sí	(0) No
1.Usted		
2. Conocido/ familiar		

60. ¿Usted cuántos amigos o familiares cercanos tiene? Entendiendo como tal a la persona que le comenta sus problemas personales o que lo ayudaría en un momento de dificultad.

____

61. ¿Usted tiene una cuenta de ahorros activa con algún banco o institución financiera?

1 [ ] Sí
0 [ ] No

62. ¿Usted gasta en loteria, chance o juegos de azar?

1 [ ] Sí → ***Indique la frecuencia**

0 [ ] No *(pase a la p63)*

	1. N° veces	2.Gasto total
1. Diario	No aplica	
2. Semanal		
3. Mensual		

CLASE MEDIA

Aplique el módulo de Clase Media sólo a Estrato 1, 2, 3 y 4 // Para Estrato 5 y 6 continúe con la pregunta 80

SERVICIOS FINANCIEROS

63. ¿Tiene alguna una tarjeta de crédito?

1 [ ] Sí → **63.1¿Cuántas?** __________ 0 [ ] No

64. ¿Tiene dificultades para pagar sus deudas?

1 [ ] Sí 2 [ ] No 3 [ ] No tiene deudas

65. ¿Cuál es el <u>principal</u> activo que hay en su hogar?

1 [ ] Carro
2 [ ] Moto
3 [ ] Vivienda
4 [ ] Computador
5 [ ] Electrodoméstico (nevera/lavadora/etc)
6 [ ] Ninguno *(pase a la p67)*
7 [ ] Otro, ¿Cuál? ______________________

66. ¿Cómo lo pagaron? (OPCIÓN MÚLTIPLE)

1 [ ] Contado
2 [ ] Crédito con familiar
3 [ ] Crédito gota a gota
4 [ ] Crédito con inst.financiera
5 [ ] No sabe/ no responde
6 [ ] Otro, ¿Cuál? ________________

67. Usted tiene un seguro de: (leer opciones) MÚLTIPLE RESPUESTA

1 [ ] Vida
2 [ ] Vivienda
3 [ ] Accidente
4 [ ] Exequial
5 [ ] Empleo
6 [ ] Ninguno
7 [ ] Otro __________

68. ¿Para usted qué es más importante?

1 [ ] Disfrutar su dinero ahora
0 [ ] Ahorrar para el futuro

CONSUMO

69. En promedio, ¿cuántas horas al día ve televisión?

____

70. En los últimos 30 días, usted ha :

	(1) Sí	(0) No	N° veces
1.Ido al cine			
2. Salido a comer			
3.Ido a la peluquería (arreglo personal)			
4.Pagado una membresía de gimnasio			NA
5.Contratado servicio doméstico			x semana

71. Durante los últimos 3 meses, usted realizó algún gasto en:

	(1) Sí	(0) No
1.Ropa para usted		
2.Ropa para niños		
3.Libros y suscripciones		
4.Remodelación de la vivienda		
5.Muebles y equipos del hogar (mesas,camas)		
6.Servicio de internet en casa		

72. Durante el último año, ¿usted se ha ido de vacaciones?

1 [ ] Sí → **72.1 ¿A dónde?** ______________________
0 [ ] No *(Pase a la p73)*

72.2 ¿Por cuánto tiempo?______________________

73. ¿Usted alguna vez ha salido del país?

1 [ ] Sí → **73.1¿Cuál es el país más lejano que conoce?**
0 [ ] No *(Pase a la p74)*

____

74. En los últimos 12 meses, ¿cuántos libros completos leyó?

____

RELACIÓN CON EL ESTADO

75.¿Usted votó en las pasadas elecciones?

1 [ ] Sí
0 [ ] No *(pase a la pregunta 77)*

76. ¿Cómo escogió por quién votar? (ÚNICA RESPUESTA)

1 [ ] Recomendación de amigo/familiar
2 [ ] Le gustó las propuestas del candidato
3 [ ] Aleatorio (azar)
4 [ ] Empatía con el candidato
5 [ ] Promesa de trabajo/Conveniencia
6 [ ] Otro, ¿Cuál? ____________________

77. Usted está de acuerdo en que el Estado le proporcione a las personas de bajos recursos: (Leer todas)

	Sí(1)	No (0)
1.Educación gratuita		
2.Salud gratuita		
3.Subsidio de servicios públicos		
4.Vivienda gratis		
5.Transferencias de dinero		

METAS ASPIRACIONALES

78. ¿En qué barrio de la ciudad le gustaría vivir? ______________________ 1 [ ] No cambiaría de barrio

79. ¿Cuál de estos factores es el más importante para su vida? (ÚNICA RESPUESTA)

LEER TODAS LAS OPCIONES

1 [ ] Tener tiempo libre
2 [ ] Tener hijos
3 [ ] Casarse
4 [ ] Tener una carrera exitosa
5 [ ] Ser rico
6 [ ] Ayudar a otros

SATISFACCIÓN CON LA CIUDAD

80. Por favor dígame en una escala de 1 a 10, qué tan satisfecho se siente con la gestión de la alcaldía en los siguientes aspectos de la ciudad, siendo 1 la peor calificación, 5 ni buena ni mala, y 10 la mejor calificación.

SEGURIDAD	SERVICIOS DE SALUD	TRANSPORTE PÚBLICO	GENERACIÓN DE EMPLEO	PARQUES Y ESPACIOS PÚBLICOS	EDUCACIÓN	SERVICIOS PÚBLICOS	TRÁFICO	BARRIO

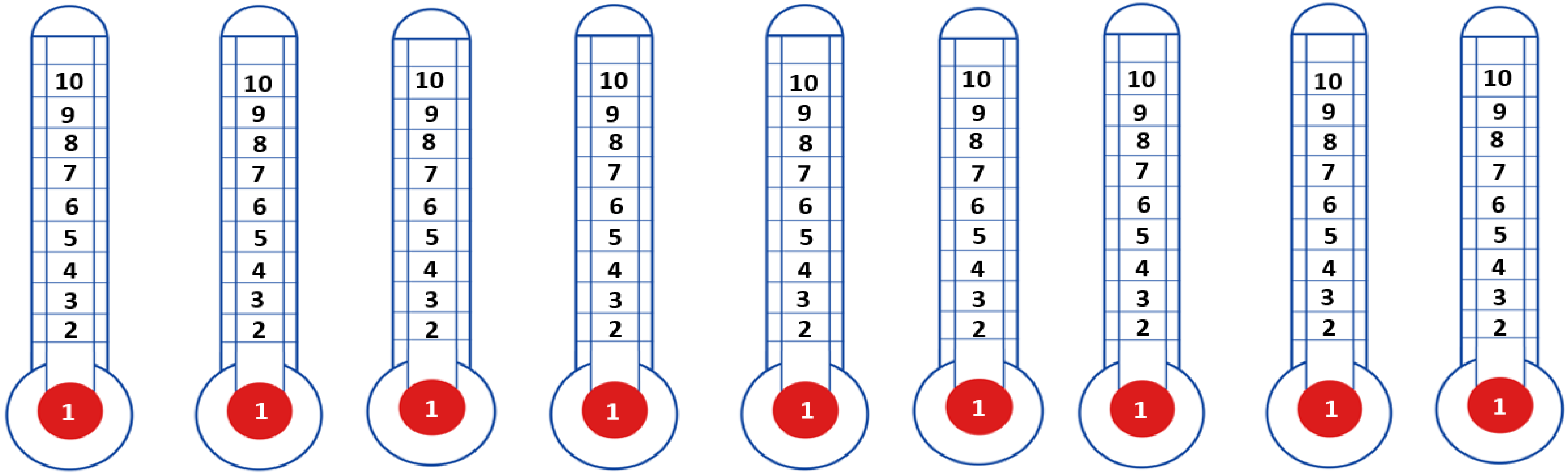

PONDERACIÓN

81. Usted acaba de calificar su satisfacción con varios aspectos de la ciudad. Le voy a entregar una tarjeta con todos esos aspectos. Dígame por favor, cuáles son los 5 factores que más afectan su satisfacción con la ciudad, siendo el primero el más importante y el quinto el menos importante, pero que igualmente influye en su satisfacción.

SATISFACCIÓN PERSONAL

82. Por favor dígame en una escala de 1 a 10, ¿qué tan satisfecho se siente con los siguientes aspectos de su vida?, siendo 1 la peor calificación, 5 ni buena ni mala, y 10 la mejor calificación.

FAMILIA	TRABAJO	VIDA SENTIMENTAL	SALUD	ECONOMÍA DEL HOGAR	INGRESO	EDUCACIÓN	LUGAR EN EL QUE VIVE
10	10	10	10	10	10	10	10
9	9	9	9	9	9	9	9
8	8	8	8	8	8	8	8
7	7	7	7	7	7	7	7
6	6	6	6	6	6	6	6
5	5	5	5	5	5	5	5
4	4	4	4	4	4	4	4
3	3	3	3	3	3	3	3
2	2	2	2	2	2	2	2
1	1	1	1	1	1	1	1

PONDERACIÓN

☐	☐	☐	☐	☐	☐	☐	☐

83. Al inicio de la encuesta usted calificó su satisfacción con la vida como ______. Le voy a entregar una tarjeta con todos los factores que acaba de calificar. Dígame por favor, cuáles son los 5 factores que más afectan su satisfacción personal, siendo el primero el más importante y el quinto el menos importante, pero que igualmente influye en su satisfación

84. ¿Hay algún factor que afecte su calidad de vida por el que no le haya preguntado?

1 ☐ Sí, ¿Cuál? ____________________
0 ☐ No

Observaciones Se refirió a Dios o a la religión para explicar su situación actual o futura: 1 ☐ Sí 0 ☐ No

____
